# Tulipa cinnabarina subsp. toprakii (Liliaceae), a new subspecies from southwestern Anatolia

**DOI:** 10.3897/phytokeys.69.9302

**Published:** 2016-08-25

**Authors:** İsmail Eker, Hasan Yıldırım, Yusuf Altıoğlu

**Affiliations:** 1 Department of Biology, Faculty of Science & Literature, Abant İzzet Baysal University, 14280 Bolu, Turkey; 2 Department of Biology, Faculty of Science, Ege University, 35100 Bornova, İzmir, Turkey

**Keywords:** New taxon, taxonomy, Tulipa, Turkey

## Abstract

A new subpecies, Tulipa cinnabarina subsp. toprakii **subsp. nov.** (Liliaceae) from Turkey is described. Diagnostic characters, descriptions, detailed illustrations, geographical distribution, conservation status and ecological observations on the new taxon are provided. It is also compared with the closely related Tulipa cinnabarina subsp. cinnabarina.

## Introduction

In Turkey, Eker et al. (2014) revised the genus *Tulipa*. According to detailed morphologic, geographic and cytotaxonomic studies of the taxa, the genus *Tulipa* was divided into two subgenera and they represented 17 species, two subspecies and two varieties (in total 19 taxa).

Ayhan Toprak, who is a biologist and “expert of plant conservation areas”, collected an interesting specimen of *Tulipa* from near Milas district in Muğla province. He sent some interesting pictures of this specimen to us for identification in 2015. In April 2016, we gathered flowering material from the natural population of this plant. As a result of our detailed morphological studies, it was concluded that the collected *Tulipa* specimens differ from all of the other *Tulipa* species by their morphological characters except of *Tulipa cinnabarina*. Although it is close to *Tulipa cinnabarina*, it is morphologically separated as subspecific rank from *Tulipa cinnabarina*.

## Materials and methods

The specimens of the new taxon were collected from their natural habitats in Turkey, and dried according to standard herbarium protocols. Voucher specimens are deposited in the herbaria; EGE and AIBU. Then, we tried to identify the specimens using the keys in the taxonomic revision of Eker et al. (2014) and the floras of neighbouring regions such as Iran (Rechinger 1990), Iraq (Wendelbo 1985), Syria, Palestine (Post and Dinsmore 1933), central Asia, Caucasus (Vvedensky 1935), and Europe (Grey-Wilson and Matthews 1980). In addition, the specimens were compared with similar specimens at international and national herbaria: AIBU, AEF, ANK, BM, ISTE, ISTF, GAZI, E, EGE, FUH, HUB, K, KNYA, NGBB and VANF. All quantitative as well as most of the qualitative characters excluding the colour features were examined in dried specimens. Measurements were made using a precise ruler under a stereo-microscope.

## Taxonomic treatment

### Tulipa cinnabarina K.Perss. subsp. toprakii

Taxon classificationPlantaeLilialesLiliaceae

Yıldırım & Eker subsp. nov.

urn:lsid:ipni.org:names:60472945-2

Figs 1 , 2

#### Diagnosis.

Tulipa cinnabarina subsp. toprakii differs from similar Tulipa cinnabarina subsp. cinnabarina by its smaller anthers, narrower outer perianth segments, outer segments with mostly blackish base or buff colored on all of outer surfaces, and smaller capsul sizes, weakly stoloniferous structure, and 2–4 leaves.

#### Type.

Turkey. Muğla: Milas, on the road of Milas to Akgedik Dam, near Yusufça Village, open slopes and in olive orchard, 457 m, 37°20'7"N; 27°52'6"E, 02 April 2016, *H.Yıldırım 3750 & Y. Altıoğlu* (holotype EGE!, isotypes AIBU!, NGBB!).

#### Description.

Plant 18.0–39.0 cm. Stem glabrous, stem width 1–2.5 mm, subterranean stem length 7.0–14.0 cm, aerial stem length 7.0–20.0 cm. Weakly stoloniferous or not. Bulb ovoid, 1.5–2.2 × 1.8–2.4 cm. Bulb neck 2.5–7.0 cm. Tunics coriaceous, dark brown, innermost tunics with a ring of short hairs around basal plate and, adpressed, pilose, bristly with longer hairs at neck, middle part glabrous. Leaves 2–4, falcate to erect-patent, lanceolate, canaliculate, glaucous, alternate or ± crowded, leaf margin membranous, glabrous or ciliate, and entire; lowest leaf 13.0–21.0 × 0.7–1.3 cm, subacute; second lowest leaf 10.0–17.0 × 0.4–0.9 cm, acute to subacute. Flower solitary, infundibular, bright dark red to orange red inside and paler red to orange red outside with mostly blackish base or buff colored on all of outer surfaces, mostly no blotch inside, rarely with a very short yellow blotch; outer tepals elliptic, narrowed at base, suacute to acute, glabrous and pubescent only at tip, 27–41 × 6–8 mm; inner tepals elliptic-obovate, narrowed at base, obtuse or obtuse-apiculate, pubescent at tip and distinctly pubescent at base, 29–44 × 10–16 mm. If it is present, outer and inner blotchs 6–10 mm in length; blotch 1/5–1/6 length of segments. Filaments blurred shades of red, orange, yellow or brown, lanceolate and pubescent at base; outer filaments 7.0–11.5 mm, inner ones 8.0–12.5 mm. Anthers 2.5–6.0 × 1.0–1.5 mm when dry, oblong, bluish-black when fresh, apiculate or not. Pollen yellow or orange. Ovary oblong, fusiform or subfusiform, pale green, yellowish-green or reddish-green when fresh, glabrous, 9.0–11.0 × 3.0–5.0 mm. Style short or obscure, 0.5–1.5 × 1.0–2 mm. Stigma pubescent, whitish-yellow or yellow when fresh, 0.5–0.6 × 1.0–1.5 mm. Capsule 1.5–2.0 × 1.0–1.3 cm, elliptic-obovoid to obovoid, glabrous; neck 0.5–1.0 mm, rib 14.0–16.0 mm, beak 2.0–4.0, apicula 0.5–1.0 mm; tepal scar 0.5–1.0 mm. Seeds numerous, flattened with rugose surface, triangular with two rounded angles to orbicular, light brown, 4.0–5.0 × 3.0–4.0 mm, winged.

**Figure 1.**
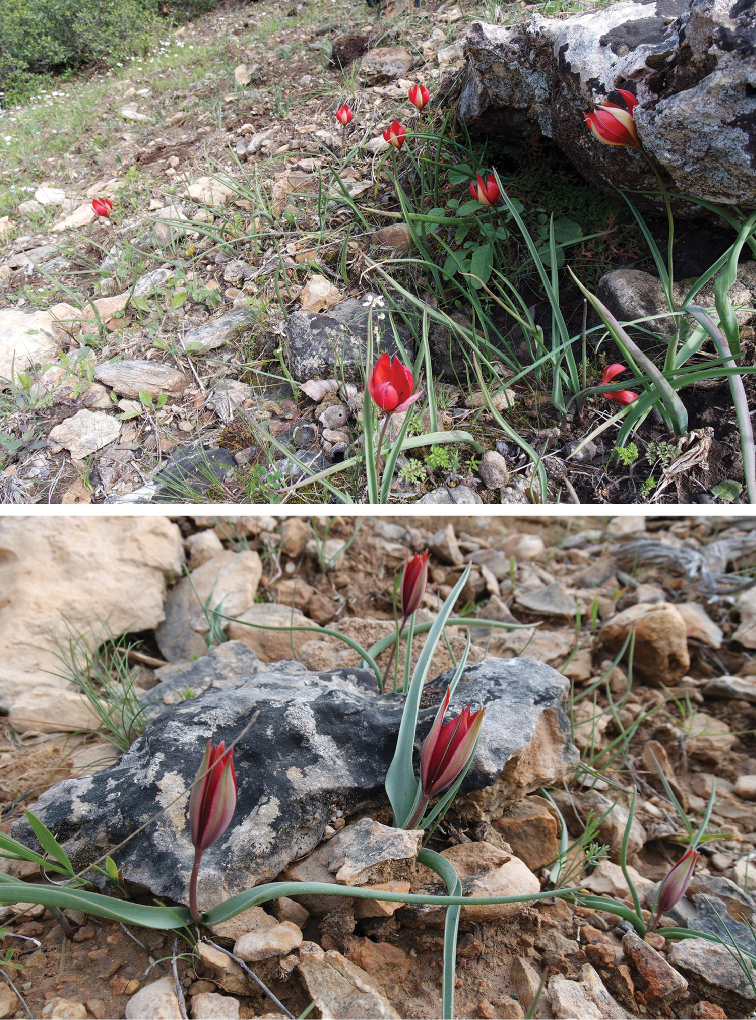
Habitat of Tulipa cinnabarina subsp. toprakii in the wild (**A–B**).

**Figure 2.**
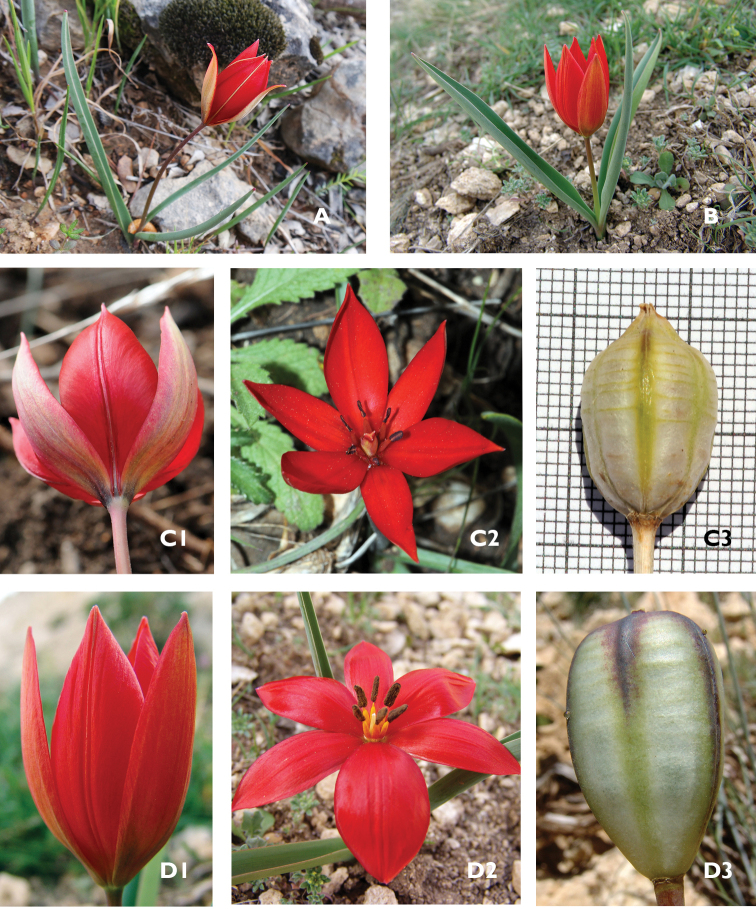
Habit of Tulipa cinnabarina subsp. toprakii (**A**) and Tulipa cinnabarina subsp. cinnabarina (**B**), Tulipa cinnabarina subsp. toprakii (**C** from *Yıldırım 3750 & Altıoğlu*), Tulipa cinnabarina subsp. cinnabarina (**D** from *Eker 2030 & 2186*), The flower from the exterior (**l**), The flower from the interior (**2**), The matured fruit (**3**).

#### Etymology.

This new subspecies is named in honour of Ayhan Toprak, who first collected the new species. The Turkish name of this species is given as “Milas Lâlesi”, according to the guidelines of Menemen et al. (2013).

#### Suggested conservational status.

The occupancy area (AOO) of Tulipa cinnabarina subsp. toprakii was calculated as 0.012 km^2^ in which about 600−750 individuals were estimated to occur. The individual of new subspecies found in *Olea europaea* L. orchard. On the other hand, overgrazing by sheep and goat herds and development of new road for mines were observed to be producing negative effects on the surroundings of the known populations, which are seriously threatened habitats of it. These strong anthropic pressures on this new subspecies are responsible for rapid habitat destruction, and they could cause a dramatic decrease of the number of reproductive individuals in the near future. Therefore, in accordance with the criteria of the IUCN (2012), Tulipa cinnabarina subsp. toprakii is here assessed as “Critically Endangered” (CR) B2ab(i,ii,iii), on account of its restricted distribution in Turkey with an inferred severe decline of the extent of occurrence, the occupancy area and quality of the habitat.

#### Distribution and ecology.

**Figure 3.**
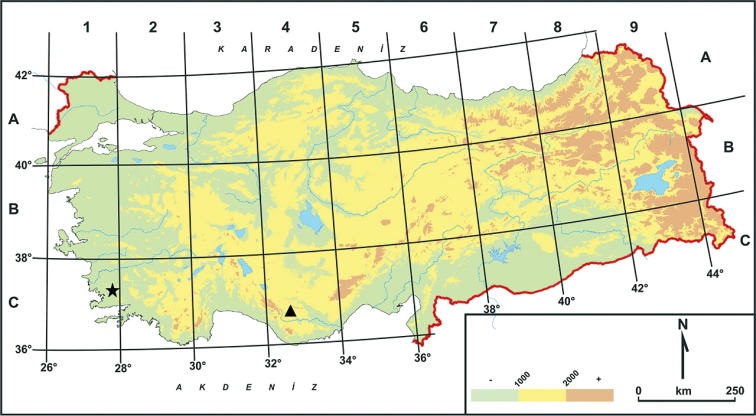
Distribution map of subspecies of *Tulipa cinnabarina*: triangle symbol is natural range of Tulipa cinnabarina subsp. cinnabarina while star symbol is natural range of Tulipa cinnabarina subsp. toprakii in Turkey.

Tulipa cinnabarina subsp. toprakii is endemic to west Anatolia (Fig. 3). It is an element belonging to the Mediterranean floristic region. It grows on calcareous soils at opening slopes and clearings in *Olea europaea* orchard. The associated species include: *Allium neopolitanum* Cyr., Anthemis cretica L. subsp. leucanthemodies (Boiss) Griersan., *Anthemis macrotis* (Rech.f) Oberpr & Voght, *Cistus creticus* L., *Fritillaria minuta* Boiss., *Melilotus indica* (L.) All., *Olea europaea*, *Ophrys iricolor* Desf., *Pinus brutia* Ten. *Ranunculus muricatus* L., *Ranunculus repens* L.

## Supplementary Material

XML Treatment for Tulipa cinnabarina K.Perss. subsp. toprakii
